# Rapid Reversible Osmoregulation of Cytoplasmic Biomolecular Condensates of Human Interferon-α-Induced Antiviral MxA GTPase

**DOI:** 10.3390/ijms232112739

**Published:** 2022-10-22

**Authors:** Pravin B. Sehgal, Huijuan Yuan, Ye Jin

**Affiliations:** 1 Department of Cell Biology and Anatomy, New York Medical College, Valhalla, New York, NY 10595, USA; 2 Department of Medicine, New York Medical College, Valhalla, New York, NY 10595, USA

**Keywords:** liquid-liquid phase separation (LLPS), biomolecular condensates, membraneless organelles (MLOs), endogenous interferon-α-induced human myxovirus resistance protein (MxA), osmoregulation, hypotonic stress, vacuole-like dilations, antiviral activity, vesicular stomatitis virus, SARS-CoV2 virus

## Abstract

We previously discovered that exogenously expressed GFP-tagged cytoplasmic human myxovirus resistance protein (MxA), a major antiviral effector of Type I and III interferons (IFNs) against several RNA- and DNA-containing viruses, existed in the cytoplasm in phase-separated membraneless biomolecular condensates of varying sizes and shapes with osmotically regulated disassembly and reassembly. In this study we investigated whether cytoplasmic IFN-α-induced *endogenous* human MxA structures were also biomolecular condensates, displayed hypotonic osmoregulation and the mechanisms involved. Both IFN-α-induced endogenous MxA and exogenously expressed GFP-MxA formed cytoplasmic condensates in A549 lung and Huh7 hepatoma cells which rapidly disassembled within 1–2 min when cells were exposed to 1,6-hexanediol or to hypotonic buffer (~40–50 mOsm). Both reassembled into new structures within 1–2 min of shifting cells to isotonic culture medium (~330 mOsm). Strikingly, MxA condensates in cells continuously exposed to culture medium of moderate hypotonicity (in the range one-fourth, one-third or one-half isotonicity; range 90–175 mOsm) first rapidly disassembled within 1–3 min, and then, in most cells, *spontaneously* reassembled 7–15 min later into new structures. This spontaneous reassembly was inhibited by 2-deoxyglucose (thus, was ATP-dependent) and by dynasore (thus, required membrane internalization). Indeed, condensate reassembly was preceded by crowding of the cytosolic space by large vacuole-like dilations (VLDs) derived from internalized plasma membrane. Remarkably, the antiviral activity of GFP-MxA against vesicular stomatitis virus survived hypoosmolar disassembly and subsequent reassembly. The data highlight the exquisite osmosensitivity of MxA condensates, and the preservation of antiviral activity in the face of hypotonic stress.

## 1. Introduction

It is now increasingly recognized that liquid-liquid phase-separation (LLPS) leads to the formation of biomolecular condensates [also called membraneless organelles (MLOs)] in the cytoplasm and nucleus of eukaryotic cells [1,2,3,4,5,6,7,8]. MLOs provide a scaffold for diverse cellular functions (e.g., cell signaling, nuclear transcription, RNA splicing and processing, mRNA storage and translation, DNA sensing, synaptic function, and mitosis) [1,2,3,4,5,6,7,8]. It is also now recognized that the replication of many viruses in mammalian cells involves LLPS condensates [e.g., in the life cycles of vesicular stomatitis (VSV), rabies (Negri bodies), influenza A, respiratory syncytial, Ebola, measles, Epstein–Barr, and SARS-CoV-2 viruses] (reviewed in [7,8]).

Four years ago, we made the discovery that human myxovirus resistance protein MxA, a 62-kDa dynamin-family large GTPase which is induced 100–300-fold in diverse cell-types by Type I IFNs (α/β families) and Type III IFNs (λ family) [9,10,11], formed phase-separated membraneless organelles (MLOs) in the cytoplasm [12,13]. MxA is a major antiviral effector of IFNs-α/β and IFN-λ against diverse RNA- and DNA-viruses [9,10,11]. Cytoplasmic MxA condensates, of gel-like internal consistency, associated with the viral nucleocapsid (N) protein of target viruses [e.g., vesicular stomatitis virus (VSV) in cells exhibiting an antiviral phenotype [13]; also see Kochs et al. 2002 for the first data for La Crosse virus N protein in *membraneless* MxA structures [14] by electron microscopy]. Remarkably, condensates formed by *exogenously* expressed GFP-tagged MxA in human Huh7 and Hep3B hepatoma cells showed rapid disassembly (within 1–2 min) in cells exposed to hypotonic medium, and rapid reassembly (within 1–2 min) into new condensate structures when cells were subsequently shifted to isotonic medium [13,15]. In the present study, we investigated whether IFN-α-induced *endogenous* MxA structures in lung-derived cells showed osmosensing properties, the underlying mechanism (s) involved, and whether osmotic disassembly/reassembly of MxA condensates affected their antiviral activity (against VSV). The data obtained highlight the rapid hypotonicity-driven disassembly and, subsequent, isotonicity-driven or even *spontaneous* reassembly of MxA condensates in lung cells. Remarkably, the antiviral activity of MxA survived osmotic cycling. Our observation that cell integrity [16,17] was required for maintenance of MxA higher-order structures in the cytoplasm places limitations on interpretation of prior data on MxA oligomerization derived from previous solution-based analyses [9,10,11,18,19,20,21,22]. More generally, the present data point to the possibility of similar limitations in biochemical studies in free solution of other proteins which form higher-order clusters and condensates in the cytosol in intact cells (e.g., the transcription factor STAT3; [16,17]).

## 2. Results

### 2.1. Properties of Endogenous MxA Condensates/Granules in Human Lung Cells

Our previous studies of cytoplasmic MxA condensates were largely carried out using GFP-tagged human MxA transiently expressed in human hepatoma (Huh7 and Hep3B) cells [13]. These GFP-MxA condensates were variably sized and shaped, showed homotypic fusion, were disassembled by 1,6-hexanediol, were membraneless by thin-section EM in a correlated light and electron microscopy (CLEM) assay, and had an internal gel-like consistency by fluorescence recovery after photobleaching (FRAP) assay [13]. Cells expressing GFP-MxA condensates showed an antiviral phenotype against VSV; in many such cells the VSV N protein associated with GFP-MxA condensates [13]. Unexpectedly, we observed that GFP-MxA condensates disassembled in 1–2 min in cells exposed to hypotonic medium (ELB; erythrocyte lysis buffer; 40–50 mOsm), and reassembled into new structures within 1–2 min of shifting cells to isotonic culture medium or phosphate-buffered saline (PBS)(~330 mOsm). Even in isotonic medium, the integrity of GFP-MxA condensate structures required an intact plasma membrane in that the addition of saponin (0.03%) to the culture medium caused rapid disassembly of the bulk of GFP-MxA condensates, occasionally leaving behind a saponin-resistant core in 10–15% of the transfected cells [13]. With increasing evidence of the involvement of IFN-α as a protective mechanism in COVID-19 caused by the SARS-CoV-2 virus, and observations showing increased levels of MxA (often referred to as Mx1 in clinical papers), in lung tissues from COVID-19 patients [23,24,25,26,27,28,29,30,31], we investigated structures formed by endogenous MxA in IFN-α-treated human lung-derived cells (the A549 adenocarcinoma line; and the SARS-CoV-2 permissive hACE2-A549 cells) under isotonic and hypoosmolar conditions.

**Figure 1:**
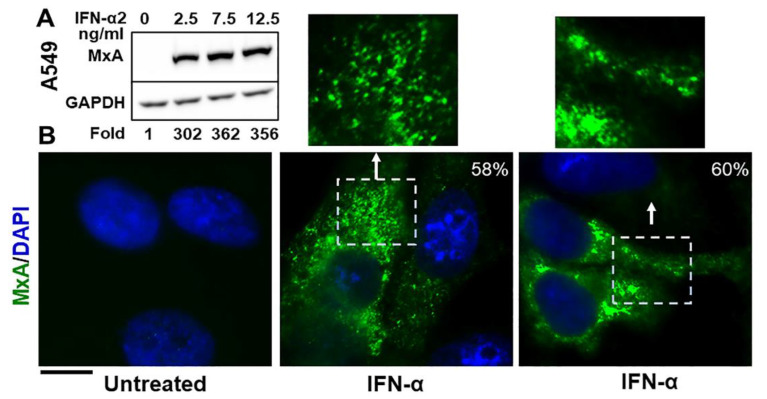
Cytoplasmic granules of endogenous MxA induced by IFN-α2 in human lung-derived A549 cells. Panel (**A**). A549 cells in culture (in 35 mm plates) were exposed to IFN-α2 at the indicated concentrations for 2 days, washed with PBS, and whole-cell extracts prepared using buffer isotonic buffer containing 0.1% SDS and 0.5% Triton X-100 (16, 42). Matching protein aliquots (30 µg) were Western blotted for MxA and GAPDH, and fold-induction of MxA by IFN evaluated using Image J (using a darker exposure to show faint MxA band in the untreated control). Panel (**B**). A549 cells in 35 mm plates without or with exposure to IFN-α2 (20 ng/mL) for 2 days, were fixed (4% PFA in PBS for 1 h at 4 °C), permeabilized (using digitonin or saponin buffer; 42), immunostained for MxA (scale bar = 10 µm). The boxed insets are also illustrated at higher magnification. Quantitation of MxA in respective images present in condensates as a percent of the total is indicated in respective panels. Briefly, images with mixed condensate and dispersed MxA were subjected to Fourier filter processing to subtract objects of small radii (3–5 pixels) in Image J. The pixel radius used for the subtraction filter was optimized to subtract all condensates from the image. MxA intensity in the residual subtracted image corresponded to the dispersed protein; subtracting this from the total intensity per cell gave the % of MxA in condensates on a per cell basis.

The Western blot data in Figure 1A confirm that exposure of A549 human lung-derived cells in culture to IFN-α2 enhanced expression of cellular MxA by more than one-hundred fold (a darker exposure was used to observe the faint band in the untreated lane; also see Figure 2A in Ref. [13] for similar data using human Huh7 hepatoma cells). By immunofluoresnece analyses this endogenous MxA protein was cytoplasmic, and was in metamorphic granular structures (Figure 1B). We used the method of Fourier transformation-based “minimum” size-filter of objects using Image J software to estimate the amount of MxA in condensates vs. diffuse in the cytosol (explained in Figure 2 in Sehgal et al., Ref. [7]; illustrated in Figure 7B below). Overall, approximately 60–70% of endogenous MxA in IFN-α-treated A549 cells was estimated to be in condensed/granular structures (Figure 1B). This is an estimate similar to that for exogenously expressed GFP-MxA (Figures 3A, 6 and 7).

**Figure 2:**
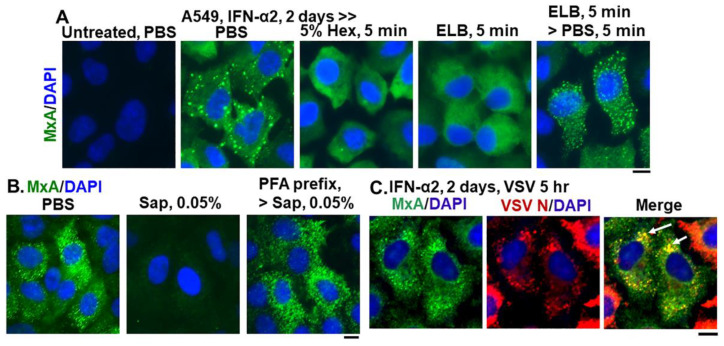
Endogenous MxA granules in IFN-α2- treated A549 cells have properties of phase-separated biomolecular condensates. Panel (**A**), A549 cells in culture (in 35 mm plates) without or with exposure to IFN-α2 (20 ng/mL) for 2 days were washed with PBS and then fixed (using in 4% PFA), or exposed to 5% 1,6-hexanediol in PBS for 5 min or to ELB for 5 min or ELB for 5 min and then PBS for 5 min prior to fixation (using 4% PFA). All cultures were then permeabilized (saponin-digitonin buffer) and immunostained for MxA. Panel (**B**), IFN-α2-treated cultures (20 ng/mL, 2 days) were fixed (4% PFA, 1 h) after a PBS wash, or were first exposed for 10 min to PBS containing 0.05% saponin, and then fixed (2% PFA for 10 min at room temperature) or first fixed with PFA (2% 10 min, room temperature), followed by saponin exposure (0.05% in PBS for 10 min). All cultures were then immunostained for MxA. Panel (**C**), IFN-α2-treated cultures (10 ng/mL, 2 days) were infected with VSV (moi > 10 pfu/cell) (13, 15) for 5 h, fixed and immunostained for MxA (green) and for VSV N (red). Arrows: cells showing condensates with both MxA and VSV N protein. All scale bars = 10 μm.

Figure 2 summarizes data showing that cytoplasmic structures derived from endogenous MxA in IFN-treated A549 lung cells, had key properties previously observed for biomolecular condensates of exogenously expressed GFP-MxA [13]. The endogenous MxA condensates were disassembled rapidly by exposure of cells to 1,6-hexanediol (5%), to hypotonic ELB and also reassembled upon subsequent exposure to isotonic PBS (Figure 2A). The endogenous MxA condensates required an intact plasma membrane for their integrity in that they were disassembled by exposure of cells to PBS containing saponin (0.05%) (Figure 2B). This observation that cell integrity was required for maintenance of MxA higher-order structures in the cytoplasm places limitations on interpretation of prior data on MxA oligomerization derived from previous solution-based analyses [9,10,11,18,19,20,21,22]. Indeed, as previously shown for stress granules and processing bodies (P-bodies) [32,33,34], prefixing of cells with 2% PFA for 10 min blocked the subsequent dissolution by saponin of MxA cytoplasmic structures (Figure 2B). Moreover, Figure 2C shows that the VSV N protein associated with condensates of endogenous MxA in A549 cells. Overall, the data in Figure 1 and Figure 2, taken together confirm that many of the properties observed previously for exogenously expressed GFP-MxA in Huh7 hepatoma cells, were also observed for cytoplasmic structures formed by endogenous MxA in IFN-α-treated A549 lung cells.

### 2.2. Accumulation of Plasma Membrane-Derived VLDs Precedes Isotonicity-Induced GFP-MxA Condensate Reassembly

**Figure 3:**
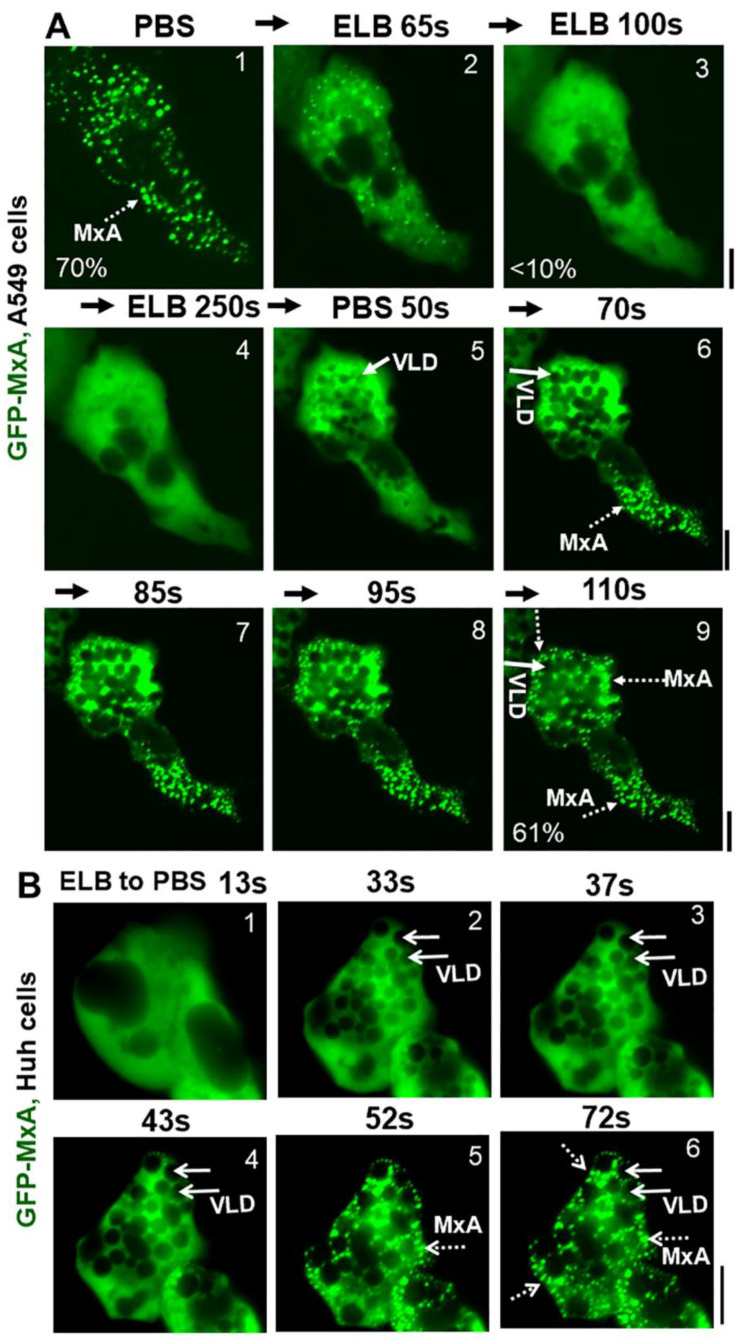
Marked accumulation of vacuole-like dilations (VLDs) in cells preceding isotonicity-driven reassembly of GFP-MxA condensates. Panel (**A**), Live-cell time-lapse images (time designated in seconds) of an A549 cell showing rapid disassembly of GFP-MxA condensates upon exposure to hypotonic ELB, and rapid reassembly of GFP-MxA into new condensates upon shift up to isotonic PBS (broken white arrows). Solid white arrows point to VLDs which accumulate in the cytoplasm prior to GFP-MxA condensate reassembly. The % numerals in respective panels are an estimate of the fraction of total cellular GFP-MxA in condensates. Panel (**B**), Live-cell time-lapse images (time designated in seconds) of a GFP-MxA expressing Huh7 cell previously shifted to hypotonic ELB (thus the GFP-MxA is dispersed) showing rapid reassembly of GFP-MxA into new condensates upon shift up to isotonic PBS (broken white arrows). Solid white arrows point to VLDs which accumulate in the cytoplasm prior to GFP-MxA condensate reassembly. Scale bars = 20 µm.

Due to the practical advantages of fluorescence microscopy studies in live cells, further studies of MxA condensate disassembly and reassembly have mainly used GFP-tagged human MxA constructs transiently expressed in A549, A549-hACE2, Huh7 and Hep3B cells at expression levels which match that of endogenous MxA in IFN-treated cells (see Figure 2A in Ref. [13] for one comparison). GFP-MxA expression in such cells led to the appearance of 70–90% of the MxA in condensates (Figure 3A). Figure 3A illustrates time-lapse images of the same A549 live cell showing the rapid disassembly (to <10% of GFP-MxA in condensates) and then reassembly of GFP-MxA condensates (up to 70% of GFP-MxA in condensates) in response to hypotonicity (ELB) and isotonicity (PBS) respectively. Figure 3B illustrates an example of GFP-MxA condensate reassembly in a live Huh7 cell following shift from ELB (hypotonic) to PBS (isotonic). Three features in the the data in Figure 3A,B and our prior data in Ref. [13] stand out: (i) that the reassembled condensates were different from the original condensates (also see time-lapse movies in Ref. [13], and Video S1 here); (ii) hypotonic disassembly and isotonic reassembly could be achieved over at least 3 cycles using 0.3 M sucrose alone to adjust tonicity (see Figure 10A in Ref. [13]); and (iii) that isotonicity-induced rapid reassembly of condensates was preceded by an even more rapid appearance of fluorescence-dark spaces vacuole-like in the cytosol. The latter vacuole-like dilated (VLD) spaces have been reported beginning over 2–3 decades ago as part of the recovery of cells from hypotonic stress and comprise the internalization of plasma membrane large vesicles [35,36,37,38,39,40,41]. It is noteworthy from the data in Figure 3 that the new reassembled GFP-MxA condensates were confined to the reduced compressed cytosolic space located between VLDs. The data are consistent with the possibility that GFP-MxA disassembly under hypotonic conditions and subsequent reassembly under isotonic conditions may represent a response to cytosol “uncrowding” and subsequent “crowding”.

**Figure 4:**
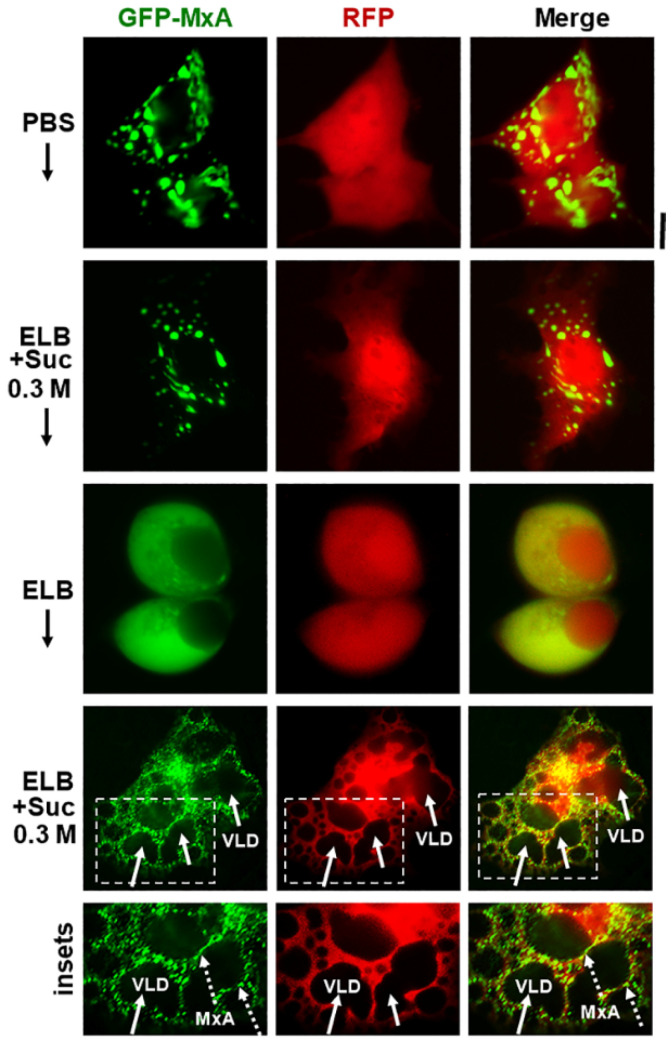
Isotonicity-driven reassembly of GFP-MxA condensates occurs in cytosolic spaces in-between crowded VLDs. Huh7 cultures were co-transfected with vectors for GFP-MxA and soluble RFP, and cells expressing both fluorescent proteins were imaged under respective changes of the culture medium as indicated (5–10 min per change). Boxed insets are shown at higher magnification in the lower-most row. Solid arrows point to VLDs, broken arrows to freshly reassembled GFP-MxA condensates. Scale bar = 10 µm.

**Figure 5:**
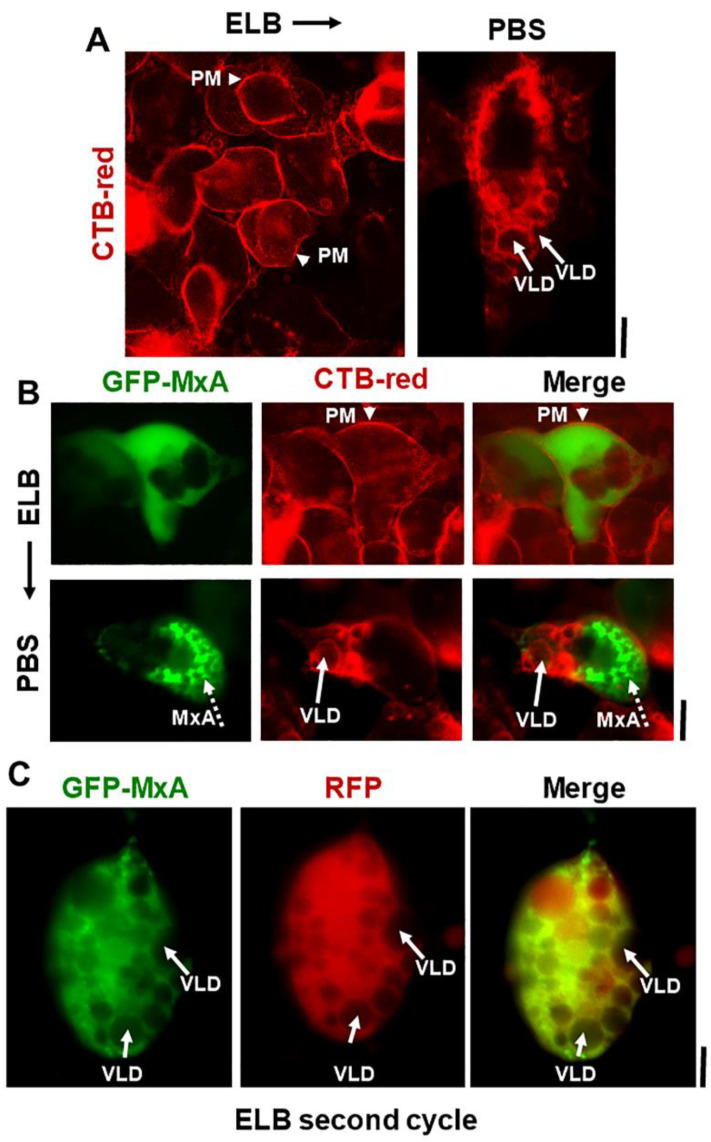
Isotonicity-driven VLDs represent internalized plasma membrane but survive a second hypotonic challenge. Panel (**A**), Live Huh7 cells swollen in hypotonic ELB were labelled with Alexafluor 594-tagged cholera toxin-red (CTB-red; 10 µM) (left panel), imaged, and were then shifted to isotonic PBS (5 min) (right panel) and reimaged. Arrow heads point to plasma membrane (PM), solid white arrows to VLDs. Panel (**B**), Live Huh7 cells expressing GFP-MxA (green) first swollen in hypotonic ELB, then labelled with CTB-red for 5 min (top row), were shifted to isotonic PBS and reimaged 2–3 min later (bottom row). Arrow head, plasma membrane (PM). Solid white arrows point to VLDs, broken white arrows to reassembled GFP-MxA condensates. Panel (**C**). Live Huh7 cell expressing both soluble RFP and newly reassembled GFP-MxA condensates (as at the end of the experiment in Figure 4; lower-most panels), were re-exposed to hypotonic ELB for a second time. Such cells, imaged 2–3 min later as in this panel, showed newly disassembled GFP-MxA despite the continued presence of a cytoplasm crowded with VLDs (solid white arrows). All scale bars = 10 µm.

Additional evidence for the above interpretation was obtained by the observations that in cells doubly expressing GFP-MxA condensates and soluble red fluorescent protein (RFP) hypotonic disassembly was accompanied by a mixing of the green and red fluorescence, and subsequent isotonicity-driven GFP-MxA condensate reassembly was confined to RFP-containing inter-VLD spaces (Figure 4). As expected, the VLD spaces were devoid of cytosolic RFP (Figure 4), consistent with the formation of VLDs by engulfment of extracellular medium during internalization of the plasma membrane [35,36,37,38,39,40,41]. Evidence that such VLDs were indeed lined by the plasma membrane was obtained using Alexafluor A594-tagged cholera toxin B (CTB) which binds the plasma membrane [42] in ELB-treated cells, and is then rapidly internalized into VLDs when cells were shifted to isotonic medium (Figure 5A,B). In cells in hypotonic ELB, the disassembled GFP-MxA was distributed in the cell contained within boundaries demarcated by CTB-red-marked plasma membrane (Figure 5B, top row). Subsequent GFP-MxA condensate reassembly took place outside of the CTB-red lined VLD spaces (Figure 5B, bottom row).

We have previously reported that ELB-driven disassembly and isotonicity-driven reassembly of GFP-MxA condensates could be repeated up to 3 times (Figures 9A and 10A in Ref. [13]). Curiously, the previous data showed that cells undergoing a second or third cycle of ELB-driven GFP-MxA disassembly continued to exhibit VLD-like dark spaces in the cytoplasm despite the disassembly and dispersal of GFP-MxA (Figures 9A and 10A in Ref. [13]). We have now confirmed the earlier findings by the observation that a second cycle of ELB-driven GFP-MxA disassembly can indeed take place in cells that continue to exhibit marked accumulation of VLDs in the cytoplasm (Figure 5C).

GFP-MxA stability in cells under isotonic conditions, condensate disassembly in cells exposed to hypotonic ELB and subsequent reassembly in cells shifted to isotonic medium was not affected by inclusion of ZnSO_4_ (200 µM) (Zn has been reported by some investigators to stabilize condensates; [43]), or CaCl_2_ (200 µM) or the calcium chelator EGTA (1 mM) in the respective buffers (data not shown). Moreover, isotonicity-driven GFP-MxA condensate reassembly was selective to MxA in that condensates of GFP-tagged SARS-CoV-2 virus nucleocapsid (N) protein did not exhibit this reassembly in isotonic PBS (Figure S1). As has been reported previously by others in other cell types [44,45,46,47], we observed that approximately 30–40% of SARS-CoV-2 N-GFP expressed in transiently transfected A549 cells was located in condensates as judged by their disassembly in 1,6-hexanediol (Figure S1A). These condensates also disassembled when cells were exposed to hypotonic ELB (Figure S1B; to <10% in condensates). However, CoV-2 GFP-N did not reassemble back into condensates in cells shifted to isotonic PBS (Figure S1B). That there was a clear distinction between condensates formed by MxA and those formed by CoV-2 N is also evident in the data in Figure S2, which shows that these two proteins formed completely distinct condensates in the cytoplasm of cells transiently co-transfected with both expression vectors (tagged either with GFP or HA in different combinations). Nevertheless, parenthetically, transient expression of GFP-MxA per se in Vero cells exhibited an antiviral activity towards SARS-CoV-2-mCherry virus (P. B. Sehgal, unpublished data).

### 2.3. Spontaneous GFP-MxA Condensate Reassembly ùnder Moderate Hypotonic Stress

ELB was used in the preceding experiments as a hypotonic buffer because it has been used over the decades by investigators to swell cells prior to mechanical breakage (such as Dounce fractionation protocols; see [16] for one example); ELB (~40–50 mOsm) is significantly hypotonic compared to isotonicity (~330 mOsm). Cells kept in ELB for an hour or more showed persistent disassembly of GFP-MxA structures and eventually lifted off the culture dish (not shown).

**Figure 6:**
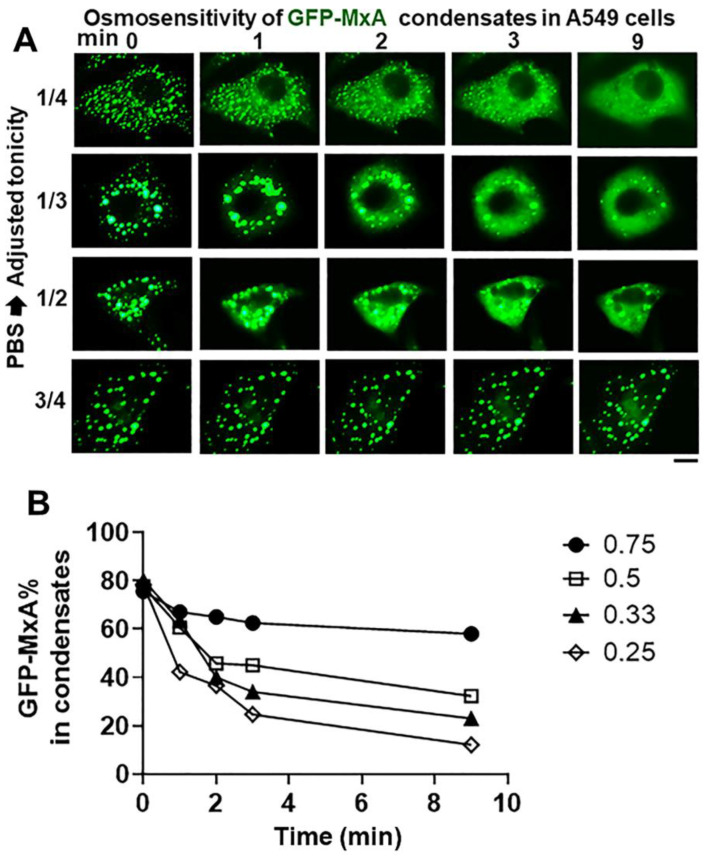
Hypotonicity-concentration dependence of the disassembly of GFP-MxA condensates. Panel (**A**), Cultures of live A549 cells expressing GFP-MxA condensates were first imaged in isotonic PBS, and then shifted to PBS diluted to 1/4, 1/3, 1/2, and 3/4 strength isotonicity using water. Individual cells in respective cultures were imaged in a time-lapse mode. Scale bar = 10 µm. Panel (**B**), Quantitation of the extent of GFP-MxA (as % of total in the cell) in condensates in the images shown in Panel (**A**). This quantitation was carried out using a Fourier filter in Image J to subtract objects of small radius (as illustrated in Figure 7B).

**Figure 7:**
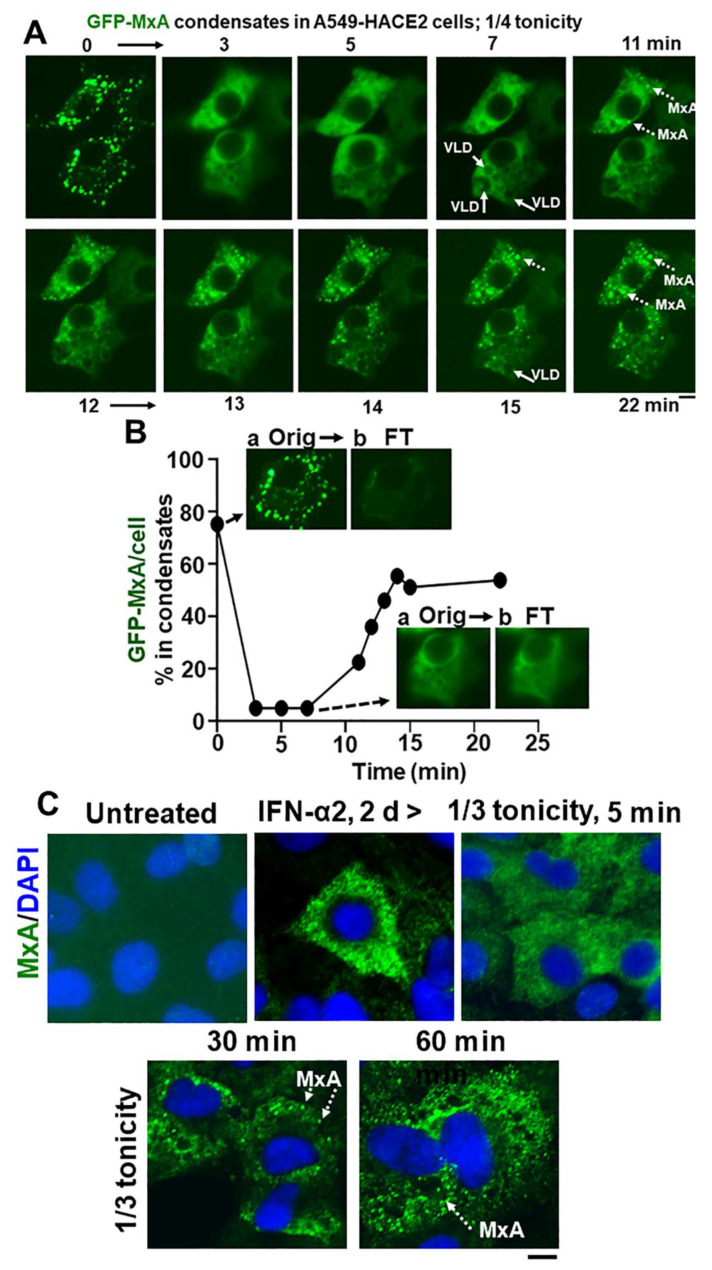
Spontaneous reassembly of MxA condensates despite continued moderate hypotonic stress. Panel (**A**), Cultures of live A549 cells containing GFP-MxA condensates were exposed to 1/4 strength hypotonic stress (1 part full culture medium diluted with 3 parts distilled water) an the cells imaged in a time-lapse mode for 25–20 min. The panel illustrates two representative cells displaying rapid disassembly of GFP-MxA condensates and then spontaneous reassembly beginning 11 min later. Solid arrows point to VLDs, broken arrows to spontaneously reassembled GFP-MxA condensates. Panel (**B**), shows quantitation of GFP-MxA in condensates (as % of total) in the lower cell in images in Panel (**A**). Insets show the process used to subtract condensates from respective images using a Fourier filter in Image J (insets a show the original image and insets b the filtered image with the condensates subtracted; these can be further quantitated using Image J to derive total and dispersed GFP values). Orig, original image; FT, filtered image. Panel (**C**), Replicate IFN-α2 (20 ng/mL, 2 days) treated cultures were exposed to 1/3 tonicity culture medium for 5, 30 and 60 min, then fixed using 4% PFA (in 1/3 tonicity PBS), followed by immunostaing for endogenous MxA. Broken white arrows point to newly spontaneously reassembled condensates of endogenous MxA. All scale bars = 10 µm.

The data in Figure 6 summarize the ability of more moderate levels of hypotonicity to disassemble GFP-MxA condensates. In this experiment, cultures first kept in isotonic PBS were exposed to hypotonic medium diluted with distilled water to give 1/4, 1/3, 1/2 and 3/4 strength tonicity (range 90–175 mOsm). The data in Figure 6A show moderate hypotonicity-dependent disassembly of GFP-MxA condensates over the next 9–10 min. Quantitation of these data was carried out using the Fourier transformation and size-filter approach (in Image J) (as illustrated in Figure 7B), The numerical estimates of the extent of disassembly (Figure 6B) show detectable disassembly in 3/4 tonicity medium, with clearcut disassembly apparent in half-tonicity medium by 2–3 min. Significant disassembly to 10–20% residual GFP-MxA in condensates was observed in cells exposed to one-third or one-fourth tonicity medium in less than 10 min (Figure 6B).

In as much as phase contrast microscopy showed that confluent A549 cultures remained intact for up to 5 h in hypotonic medium in the range 1/4, 1/3, 1/2 isotonicity, we extended the time of observation of GFP-MxA structures in cells exposed to moderate hypotonic medium for up to 60 min. Much to our surprise, in such cells, the disassembled GFP-MxA *spontaneously* reassembled into new condensates by 7–15 min (Figure 7A,B). This spontaneous reassembly was preceded by the appearance of VLDs in such cells (Figure 7A). Quantitation of the extent of GFP-MxA in condensate vs. diffuse state in a cell was carried out using the Fourier-based “minimum” filter in Image J to subtract small objects such as condensates from images (Figure 7B). Video S1 shows time-lapse images (5 sec/frame) of the disassembly of GFP-MxA condensates in an A549-hACE2 cell shifted to one-third isotonic medium, and the subsequent spontaneous reassembly of GFP-MxA into new condensates in such cells 10–15 min later. The live-cell movie again shows the appearance of the dark GFP-free VLD spaces prior to the reassembly of GFP-MxA into condensates. This spontaneous reassembly of MxA condensates under moderate hypotonicity conditions was also confirmed for endogenous MxA condensates/granules (Figure 7C).

**Figure 8:**
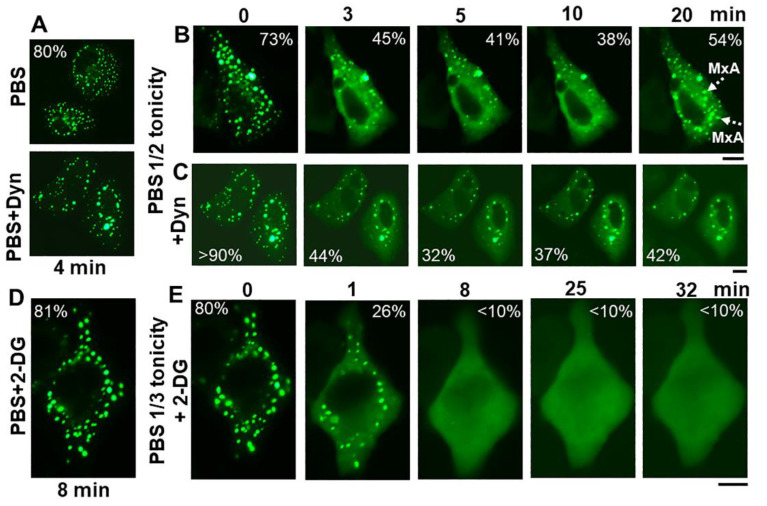
Spontaneous reassembly of GFP-MxA condensates is inhibited by dynasore and 2-deoxyglucose (2-DG). Panels (**A**–**C**), Cultures of A549 cells expressing GFP-MxA were shifted to PBS and then exposed dynasore (Dyn; 100 µM in PBS) for 4 min, followed by a shift to one-half strength PBS without or with inclusion of dynasore (100 µm). Panel illustrates a compilation of respective time-lapse images; numerals in each panel indicate % of GFP-MxA in condensates (evaluated as per the Fourier filter method depicted in Figure 7B). Broken white arrows point to newly spontaneously reassembled GFP-MxA condensates. Panels (**D**,**E**), Cultures were treated with PBS supplemented with 2-DG (10 mM) for 8 min, and then shifted to one-third strength PBS with inclusion of 2-DG (10 mM). All scale bars = 10 µm.

The discovery of *spontaneous* reassembly of GFP-MxA condensates in cells under moderate hypotonic conditions, allowed for testing the contribution of various biochemical mechanisms towards GFP-MxA condensate formation. Figure 8 summarizes an experiment in which dynasore (100 µM) was observed to reduce the extent of reassembly—an observation consistent with the possibility that plasma membrane internalization (such as to form VLDs) could drive MxA reassembly.

The inclusion of 2-deoxyglucose (2-DG; 10 or 20 mM; this inhibits ATP generation [48]) in full-strength PBS did not affect GFP-MxA condensate structures (Figure 8D). The inclusion of 2-DG in 1/3rd strength PBS did not affect GFP-MxA disassembly (Figure 8E), However, 2-DG inhibited condensate reassembly in 1/3rd tonicity medium (Figure 8E), suggesting that this spontaneous reassembly was energy dependent. This approach (Figure 8) allows for further investigations of inhibitors which stabilize condensates [49,50] or inhibit various aquaporin water channels in the plasma membrane [51].

### 2.4. Antiviral Activity of GFP-MxA against VSV Survives Osmotic Disassembly/Reassembly

We monitored the replication of VSV at the single-cell level (by immunofluorescence for VSV N) in cultures transiently transfected with a GFP-MxA construct and then with VSV at moi >10 pfu/cell followed by fixation 4 or 5 h after infection which is near the peak of VSV N expression in infected cells [13,52,53]. Because we wished to evaluate the effect of disassembly and reassembly of GFP-MxA condensates lasting several minutes to a few hours on VSV replication soon after initiation of the infection, the approach used required a single-cycle replication assay under conditions in which >90% of cells in a culture were infected (moi >10 pfu/cell), and not a low-multiplicity multiple-cycle virus assay. Moreover, VSV N protein expression was evaluated by immunofluorescence (in red) at the single-cell level in GFP-MxA expressing and GFP-MxA-free cells located side-by-side in the same culture. Thus, all cells (without or with GFP-MxA expression) were exposed to the same virus and the same osmotic manipulation in the same culture, and thus the cells without GFP-MxA expression (by subsequent fluorescence microscopy in green) served as controls for the effect (if any) of osmotic manipulation on VSV replication per se (quantitated by red immunofluoresence).

Data in Figure S3, left most culture, validate this approach–GFP-MxA expressing cells and showed a marked reduction in VSV N immunofluorescence (also see [13,15] for additional examples). Greater than 90% of all cells expressing GFP-MxA showed marked reduction in VSV-N expression (also see quantitative data in Figure 12A in Ref. [13]) The possibility that hypotonicity-induced GFP-MxA disassembly might affect antiviral activity was investigated in two ways. In one approach, cells the infected GFP-MxA expressing cultures were subjected to a 5 min cycle of ELB and then returned to full culture medium at 1, 2 or 3 h after infection, and the antiviral activity assessed at the single-cell level at 4 h after infection (Figure S3). In the second approach, cultures were switched to one-third tonicity medium 45 min after the start of infection, fixed at 5 h after start of infection, and expression of VSV N evaluated in GFP-MxA-negative and in GFP-MxA positive cells. In both instances, the disassembly and reassembly of GFP-MxA (as expected) was verified by live-cell imaging of the same cultures (not shown).

The data in Figure S3, middle and right-most cultures, show that one 5-min cycle of disassembly/reassembly of GFP-MxA at 1 h or 3 h after the beginning of VSV infection had little effect on the antiviral activity. In a similar experiment, one 5-min cycle of disassembly/reassembly at 2 h after the start of infection also had little effect on antiviral activity (not shown).

**Figure 9:**
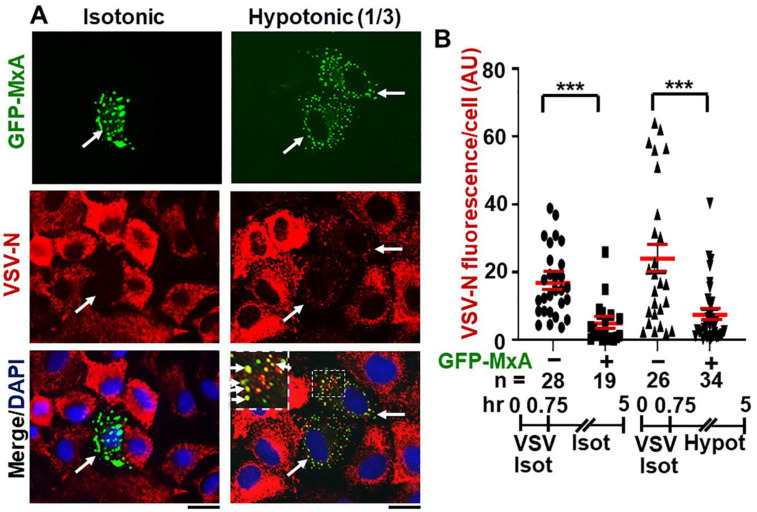
Antiviral activity of GFP-MxA against VSV survives hypotonic stress. Panel (**A**), A549 cells were transfected with pGFP-MxA and 2 days later were exposed to VSV (moi >10 pfu/cell) for 45 min in isotonic serum-free DMEM (Isot). Cultures were then washed and maintained for the next 4.25 h in regular medium (Isot) or one-third strength medium (Hypot). The cultures were then fixed and imaged for GFP-MxA (green) and for VSV-N (immunostained in red). Solid white arrows: GFP-MxA expressing cells with reduced VSV-N. Inset: GFP-MxA condensates which incorporate VSV N (white arrows). Scale Bar = 20 μm. Panel (**B**), quantitation of VSV N per cell in GFP-MxA positive (+) or negative (−) individual cells in the same culture kept without or with one-third hypotonic stress in the experiment in Panel (**A**). n = number of cells evaluated per group in this experiment; horizontal red lines within each group indicate Mean ± SE. Statistical significance was evaluated using one-way ANOVA (Kruskal–Wallis with Dunn’s post hoc test for multiple comparisons); *** *p* < 0.001.

Figure 9A,B shows that continued maintenance of cells in one-third isotonic medium for >4 h also had little effect on the antiviral activity of GFP-MxA. Remarkably, many cells showed the co-localization of VSV-N with the reassembled GFP-MxA condensates (Figure 9A). Overall, the antiviral activity of GFP-MxA against VSV largely survived this edema-like hypoosmolar stress.

## 3. Discussion

**Figure 10:**
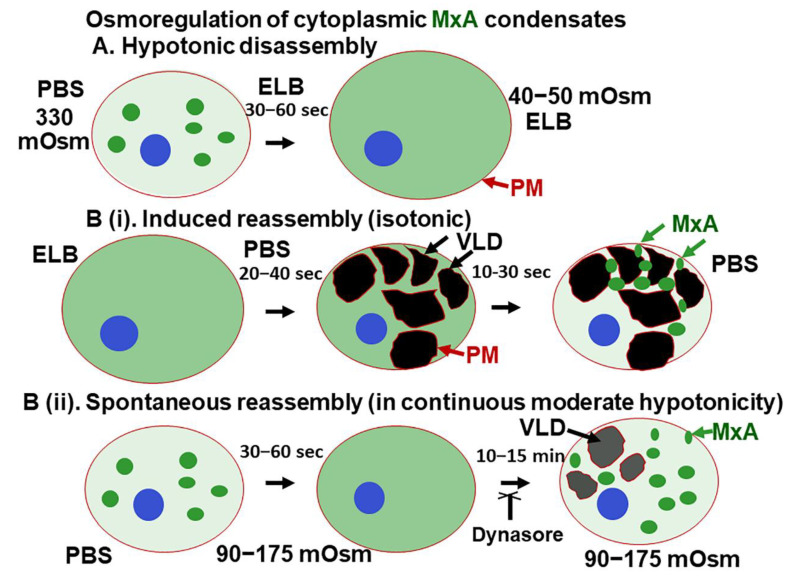
Schematic summary of osmoregulation of cytoplasmic MxA condensates. ELB, hypotonic erythrocyte lysis buffer; PBS, phosphate-buffered saline; PM, plasma membrane (displayed using CTB-red as in Figure 5); VLD, vacuole-like dilatation.

The present study highlights a dramatic property of phase-separated biomolecular condensates of the human antiviral MxA protein—rapid reversible sensitivity to cellular responses to hypoosmolar changes in the extracellular fluid (Figure 10). Lung- and liver-derived cells exposed to even mild hypotonicity exhibited rapid disassembly of MxA condensates within 1–3 min. Remarkably, the dispersed MxA reassembled into new condensates either upon subsequent shifting of cells to isotonic medium (induced reassembly) or even spontaneously in cells maintained in moderately hypoosmolar conditions (Figure 10B). Isotonicity-driven or even spontaneous MxA condensate reassembly was preceded by the accumulation of plasma membrane-lined VLDs in the cytoplasm (Figure 10). The biochemical basis for a relationship between VLD dynamics and MxA condensate formation included “crowding” of the liquid phase of the cytosol by VLDs, and the inhibition of reassembly by dynasore (evidencing dependence on membrane internalization) and by 2-deoxyglucose (evidencing dependence on ATP). Importantly, the antiviral activity of MxA against VSV, a virus which replicates exclusively in the cytoplasm, survived osmotic disassembly/reassembly. As a technical issue, the observation that cell integrity was required for maintenance of MxA higher-order structures in the cytoplasm places limitations on interpretation of prior data on MxA oligomerization derived from numerous previous solution-based analyses [18,19,20,21,22].

We previously reported that endogenous MxA induced by exposure of primary human pulmonary arterial endothelial cells in culture to IFN-α was present in the cytoplasm in granular structures [12,54]. In the present study, we provide evidence showing that cytoplasmic structures produced by endogenous MxA induced by IFN-α in lung derived cells had properties similar to biomolecular condensates formed by exogenously expressed GFP-MxA—disassembly by 1,6-hexanediol, disassembly in cells exposed to hypotonic ELB, with reassembly upon subsequent isotonic shift; and incorporation of the viral (VSV) nucleoprotein in MxA condensates (Figure 2). An intact plasma membrane was necessary to maintain MxA condensate integrity. Puncturing the plasma membrane with saponin at a low concentration (0.03–0.05%) led to disassembly of almost all the condensates produced by endogenous MxA (Figure 2B), and most of those produced by exogenously expressed GFP-MxA [13]. With both endogenous MxA (Figure 2B) and GFP-MxA [13], saponin caused a net loss of MxA from the cytoplasmic compartment (into the culture medium). However, as with data previously obtained by other investigators in studies of stress granules and P-bodies [32,33,34], pre-fixing of cells with paraformaldehyde (2% at room temperature for 10 min) stabilized MxA condensates to a detergent exposure (Figure 2B). Thus, prior attempts to identify cellular proteins which associated with MxA using protocols involving cell breakage likely provide incomplete answers [9,10,11,18,19,20,21,22].

MxA is a dimeric protein which readily multimerizes into larger linear and ring-shaped assemblies in solution [9,10,11,18,19,20,21,22]. The globular GTPase is hinged to a stalk region consisting of 4 α-helicies linked one to the other by disordered loops (L1 to L4). The L4 loop contains a stretch of 4 Lys residues important for multimerization and antiviral activity. It is already clear that MxA can readily form protein clusters (higher-order oligomers) in solution [18,19,20,21,22]. Although the L4 loop represents an intrinsically disordered region, the structural bases for the formation of phase-separated MxA condensates are incompletely understood.

The salt-dependence of MxA oligomerization reported in previous cell-free solution-based observations [18,19,20,21,22] are the reverse of what we now report for MxA in intact cells. Specifically, Haller and colleagues reported that in cell-free solutions recombinant MxA formed large linear and ring-shaped oligomers in low salt conditions (50 mM NaCl); these persisted at 150 mM NaCl but were disrupted into small structures at 300 mM NaCl [18,19,20,21]. Oligomerization was further enhanced in the presence of GDP-Al fluoride or GMP-PCP [18,19,20,21]. Previous studies in cell-free solutions emphasizing MxA oligomerization under low-salt conditions (50 mM NaCl) are consistent with electrostatic interactions driving oligomerization in solutions of recombinant MxA in such assays. However, in intact cell studies, we now report that hypotonic exposure of cells disrupts MxA condensates, while isotonic (and even hypertonic) exposure of cells reassembles condensates. Recent biophysical insights [34,55,56] which suggest a continuum of “fuzzy interactions” (low-affinity with high/variable stoichiometry) depending on the cytosol context between protein monomers with a multivalent tendency, with or without the participation of other protein components or nucleic acid polymers, to generate “protein clusters” extending up in size to the formation of higher-ordered structures and even phase-separated condensates, may apply to human MxA in the cell cytoplasm. Our observations that puncturing the cellular plasma membrane using low-concentration of saponin (0.03 to 0.05%) leads to a major loss of condensates formed by GFP-MxA (13) and endogenous MxA (Figure 2B) suggests that the behavior of recombinant MxA in free solution (as monomers, dimers and tetramers and even assembly of higher-order oligomers in low-salt solutions) as elucidated over the last two decades [9,10,11,18,19,20,21] may not be fully applicable to the physiological behavior of MxA protein clusters/condensates in the protein-crowded cytosol of the intact cell (disassembly in low-salt media). This limitation of studies of protein oligomerization in free solution is likely applicable to other proteins—our data suggest that the transcription factor STAT3 (92 kDa) also forms variably sized clusters (of size in the range from 200 KDa to 2 MDa) in the cytosol and can also form phase-separated cytoplasmic and nuclear condensates [16,17,57].

The dramatic accumulation of VLDs in the cytoplasm of cells shifted from hypotonic conditions to isotonic conditions has been recognized in numerous studies over the last 3 decades [35,36,37,38,39,40,41]. In particular, Sheetz and colleagues have investigated the generation of mechanical plasma membrane tension in mast cells and neuronal cells subjected to tonicity changes [36,39,40]. A decrease in plasma membrane tension (such as when swollen cells are shifted to isotonic medium) leads to marked endocytosis as the cell readjusts its surface membrane area. The endocytosed structures formed included large mesoscale vacuole-like dilations (VLDs) which crowd the cytosolic space. It is noteworthy that in the experiments shown in Figure 3 and Video S1, VLD formation preceded the reassembly of GFP-MxA condensates. Thus, cytosolic crowding due to this accumulation of VLDs may represent a mechanism contributing to MxA phase separation and condensate formation. That dynasore, which inhibits clathrin-mediated endocytosis, showed an inhibitory effect on MxA condensate reassembly is consistent with a role of plasma membrane internalization in the osmo-mechanical regulation of MxA condensate formation. This mechanism was selective in that there was little reassembly of condensates of the nucleocapsid protein (N-GFP) of SARS CoV-2 virus when cells were cycled from hypotonic conditions to isotonic medium (Figure S1). Thus, different condensates even in the same cell can respond differently to cellular events during osmotic stress.

The formal relationship between VLD accumulation and “crowding” in the biophysical sense of removal of water molecules from the liquid phase of the cytosol remains unclear. The possibility that additional biochemical mechanisms participate in the osmoregulation of MxA condensate disassembly/reassembly remains open [58]. Indeed, MxA has been shown to interact physically with the TRPC-family of stretch receptors (TRPC-1, -3, -4, -5, -6 and -7) and to regulate Ca^++^ signaling [59].

Hyperosmotic stress (HOPS) also promotes reversible condensate formation in human cells by multivalent proteins, especially those associated with P bodies [60,61,62]. The trimeric protein DCP1A, expressed in human cell lines, underwent rapid and reversible condensate formation when cells were shifted from isotonic medium (150 mM NaCl) to hypertonic medium (300 mM NaCl). More generally, proteins with a self-interacting valency ≥2 underwent HOPS [60]. The functional consequences included transcription termination defects during osmotic stress because of condensate formation by transcription accessory proteins such as CPFS6 [60]. In the case of GFP-MxA, hyperosmotic medium made the condensates more compact and cigar-like in intact cells [13]. In contrast, Haller and colleagues have reported that in cell-free solutions 300 mM NaCl caused the truncation of MxA oligomers into smaller structures [18,19,20].

The relationships between MxA condensate formation and antiviral activity appear to be complex. The structural basis for the antiviral effect of human MxA against both the myxoviruses such as FLUVA (which include a nuclear 5′ cap-snatching step in their replication) and rhabdoviruses such as VSV (which replicate entirely in the cytoplasm) are incompletely understood [9,10,11,63]. MxA is customarily considered to inhibit early transcription of the incoming virus in the cytoplasm (such as when targeting VSV) as well as later steps in virus replication (such as when targeting FLUAV). Cell-free experiments confirm the ability of recombinant MxA to inhibit viral transcriptional activity (reviewed in [9,10,11,63,64]). However, it is unclear whether or how monomeric, dimeric or higher-order structures of MxA associate with viral components in mediating this inhibition in intact cells. The present quantitative data show a dynamic equilibrium between MxA amounts in cytoplasmic condensates and in the dispersed mode in IFN-α-stimulated cells or in cells expressing GFP-MxA with 60–80% of the MxA in condensates. These data are consistent with the presence of least some dispersed MxA at all times in most cells. Previous mutational studies of human MxA showed that the GTPase activity was required for most of its antiviral activity (except that against hepatitis B virus) (reviewed in [9,10,11,63,64]). Inspection of data in the literature also reveals that MxA mutants lacking GTPase activity (and thus antivirally inactive), can still form cytoplasmic condensates (20, 21). Mutations that cause dispersal of MxA in the cytoplasm (e.g., the D250N mutant) lacked antiviral activity [21,63]. However, the R645 point mutant of MxA, which formed larger *cytoplasmic* granules, had the unusual property of inhibiting FLUAV but not VSV, even though the wild-type MxA showed antiviral activity towards both viruses [9,10,11]. The subcellular compartment within which respective Mx proteins reside (cytoplasm or nucleus) also affect their antiviral spectrum [15,63]. As one comparison, cytoplasmic human MxA and nuclear murine Mx1 both inhibit FLUAV replication (which has replication-critical steps in both the cytoplasm and nucleus), but only cytoplasmic human MxA but not nuclear murine Mx1 inhibits VSV (which replicates entirely in the cytoplasm) [9,10,11,15].

To conclude, as with exogenously expressed GFP-MxA, IFN-α-induced *endogenous* MxA structures in human cells were confirmed to be cytoplasmic phase-separated biomolecular condensates. Indeed, condensates formed by both endogenous MxA and exogenously expressed GFP-MxA showed rapid disassembly in cells subjected to hypotonic stress, and, subsequent, isotonicity-driven or even *spontaneous* reassembly of MxA into new condensates. Mechanistically, this MxA condensate reassembly was preceeded by crowding/compression of the cytosolic space by plasma membrane-lined vacuoles (VLDs). The data also emphasize the preservation of antiviral activity of MxA in the face of hypotonic stress.

## 4. Materials and Methods

### 4.1. Cells and Cell Culture

Human hepatoma cell line Huh7 was a gift from Dr. Charles M. Rice, The Rockefeller University. New York, NY, USA. Human Hep3B, and A549 cells were obtained from the ATCC (Manassus, VA, USA). Additional aliquots of the A549 cells and its derivative line A549-hACE2 was obtained from BEI Resources/ATCC (Manassus, VA). The respective cell lines were grown in DMEM (Corning Cat. No. 10-013-CV, with glutamine, Na-pyruvate and high glucose) supplemented with 10% *v*/*v* fetal bovine serum (FBS) in T25 flasks [13,15]. For experiments, the cells were typically plated in 35 mm dishes without or with cover-slip bottoms [13,15]. Recombinant human IFN-α2 was purchased from BioVision (Milpitas, CA, USA) and typically used at 10–20 ng/mL for 2 days. Whole-cell extract and Western blot analyses for MxA were carried out as previously reported [12,13].

### 4.2. Plasmids and Transient Transfection

The GFP (1-248)-tagged full-length human MxA was a gift from Dr. Jovan Pavlovic (University of Zurich, Switzerland) [65]; the expression vector for the HA-tagged full-length human MxA was a gift from Dr. Otto Haller (University of Freiburg, Freiburg, Germany) [66].; the HA and GFP tags were located on the N-Nterminal side of the Mx coding sequence. The expression vector for soluble RFP was a gift from Dr. Jason Lee (Baylor University School of Medicine, Houston, TX, USA). Codon-optimized expression vactors for HA-tagged and GFP-tagged nucleocapsid (N) protein of SARS-CoV-2 virus were purchased from Sino Biologicals US Inc, Wayne, PA, USA). Transient transfections were carried out using just subconfluent cultures in 35 mm plates using DNA in the range of 0.3-2 µg/culture and the Polyfect reagent (Qiagen, Germantown, MD, USA) and the manufacturer’s protocol (with 10 µL Polyfect reagent per 35 mm plate).

### 4.3. VSV Stock and Virus Infection

A stock of the wild-type Orsay strain of VSV (titer: 9 × 10^8^ pfu/mL) was a gift of Dr. Douglas S. Lyles (Dept. of Biochemistry, Wake Forest School of Medicine, Winston-Salem, NC, USA). Single-cycle virus infection studies at high multiplicity (moi >10 pfu/mL) were carried out essentially as described by Carey et al. [12] as summarized in Davis et al. [13] and in Sehgal et al. [15]. Briefly, A549 cultures (approx. 2 × 10^5^ cells per 35 mm plate), previously transfected with the pGFP-MxA expression vector (1–2 days earlier), were replenished with 0.25 mL serum-free Eagle’s medium and 10–20 µL of the concentrated VSV stock added (corresponding to MOI >10 pfu/cell). The plates were rocked every 15 min for 45 or 60 as indicated in respective experiments followed by addition of 2.5 mL of full culture medium. For the experiment shown in the cultures were subjected to a 5 min cycle of GFP-MxA condensate disassembly in ELB (verified by live-cell microscopy) and then returned to full culture medium till 4 h post-infection (pi). For the experiment in Figure 9, cultures infected for 45 min with VSV in isotonic DMEM, were shifted to full culture medium or one-third strength culture medium for another 4.25 h. Cultures were fixed in 4% PFA in isotonic PBS or one-third strength PBS (1 h at 4 °C immunostained for VSV nucleocapsid (N) protein using an mAb provided by Dr. Douglas S. Lyles (mAb 10G4). N-protein immunofluorescence (in red) in GFP-positive (nuclear or cytoplasmic) and negative cells was quantitated on a per cell basis as summarized in Davis et al. [13] and Sehgal et al. [14] and expressed in arbitrary units (AU) as integrated intensity/cell.

### 4.4. Live-Cell Fluorescence Imaging

Live-cell imaging of GFP-MxA structures in transiently transfected cells was carried out in cells grown in 35 mm plates using the upright the Zeiss AxioImager 2 equipped with a warm (37 °C) stage and a 40× water immersion objective, and also by placing a coverslip on the sheet of live cells and imaging using the 100× oil objective (as above) with data capture in a time-lapse or z-stack mode (using Axiovision 4.8.1 software) [13].

### 4.5. Phase Transition Experiments

Live GFP-MxA expressing cells in 35 mm plates were imaged using a 40× water-immersion objective 2–3 days after transient transfection in growth medium or serum-free DMEM medium or in phosphate-buffered saline (PBS). After collecting baseline images of Mx condensates (including time-lapse sequences), the cultures were exposed to 1,6-hexanediol (5% *w*/*v*) in PBS, or to hypotonic buffer (ELB; 10 mM NaCl, 10 mM Tris, pH 7.4, 3 mM MgCl_2_) and live-cell time-lapse imaging continued [13]. After approximately 5–10 min the cultures exposed to hypotonic ELB were replenished with isotonic phosphate-buffered saline (PBS) and imaged for another 5–10 min. Time lapse images were also collected upon exposure of the cell cultures to different hypotonic buffers and treatments. Time lapse movies shown in Video S1 was imaged at one frame/5 s.

### 4.6. Immunofluorescence Imaging

Typically, the cultures were fixed using cold paraformaldehyde (4%) for 1 h and then permeabilized using a buffer containing digitonin or saponin (50 µg/mL) and sucrose (0.3M) [13]. Single-label and double-label immunofluorescence assays were carried out using antibodies as indicated, with the double-label assays performed sequentially. Fluorescence was imaged as previously reported [13,15] using an erect Zeiss AxioImager M2 motorized microscopy system with Zeiss W N-Achroplan 40×/NA0.75 water immersion or Zeiss EC Plan-Neofluor 100×/NA1.3 oil objectives equipped with an high-resolution RGB HRc AxioCam camera and AxioVision 4.8.1 software in a 1388 × 1040 pixel high speed color capture mode. Colocalization analyses were carried out using Image J software (Fiji) expressed in terms of Pearson’s correlation coefficient R.

### 4.7. Quantitation of Relative Amounts of MxA in Condensates vs. Dispersed State in a Cell

The procedure used to quantitate the relative amounts of MxA in the condensed vs. dispersed state on a per cell basis, as explained in Figure 2 of Ref. [7], is illustrated in Figure 7B. Briefly, images with mixed condensate and dispersed MxA were subjected to Fourier filter processing to subtract objects of small radii (2–4 pixels) in Image J. The pixel radius (in the range 2–4 pixels) used for the subtraction was optimized to subtract all condensates from the image. MxA intensity in the residual subtracted image corresponded to the dispersed protein; subtracting this from the total intensity per cell gave the % of MxA in condensates on a per cell basis (see Figure 7B).

### 4.8. Antibody Reagents

Rabbit pAb to human MxA (H-285) (ab-95926) was purchased from Abcam Inc. (Cambridge, MA, USA); Mouse mAb to the VSV nucleocapsid (N) designated 10G4 was a gift from Dr. Douglas S. Lyles (Wake Forest School of Medicine, Winston-Salem, NC, USA). Rabbit mAb to glyceraldehyde-3-phosphate dehydrogenase (GAPDH; 14C10; number 2118) was obtained from Cell Signaling (Danvers, MA, USA), Anti-HA murine mAb (Cat. No. 100028-MM10) was purchased from Sino Biologicals US Inc. (Wayne, PA, USA). Respective AlexaFluor 488- and AlexaFluor 594-tagged secondary donkey antibodies to rabbit (A-11008 and A-11012) or mouse (A-21202 and A-21203) IgG were from Invitrogen Molecular Probes (Eugene, OR, USA). Alexafluor-594 tagged recombinant cholera toxin subunit B (CTB-red) was also purchased from Invitrogen Molecular Probes and used as per manufacturer’s recommendations (10 µM final concentration).

### 4.9. Statistical Testing

This was carried out (as in Figure 9B and Figure S3) using non-parametric one-way ANOVA (Kruskal–Wallis) with Dunn’s post hoc test for multiple comparisons.

## Data Availability

All data are available within the manuscript.

## Supplementary Materials

The following supporting information can be downloaded at: https://www.mdpi.com/article/10.3390/ijms232112739/s1.

## Abbreviations

2-DG, 2-deoxyglucose, CLEM, correlated live-cell fluorescence and electron microscopy; ELB, hypotonic “erythrocyte” lysis buffer; EM, thin-section EM; FLUAV, influenza A virus; FRAP, fluorescence recovery after photobleaching assay; GAPDH, glycerandehyde 3-phosphate dehydrogenase; LLPS, liquid-liquid phase separation; MLO, membraneless organelle; Mx, myxovirus resistance protein; MuMx1, murine Mx1; MxA or HuMxa, human MxA; N protein, nucleocapsid protein; P bodies, cytoplasmic processing bodies; pi, post-infection; PM, plasma membrane; TEA, tetraethylammonium; TGT-020, *N*-1,3,4-thiadiazol-2-yl-3-pyridinecarboxamide; VLD, vacuole-like dilation; VSV, vesicular stomatitis virus.
