# Supplementary material for: Rapid Reversible Osmoregulation of Cytoplasmic Biomolecular Condensates of Human Interferon-α-Induced Antiviral MxA GTPase
Source: Int J Mol Sci. 2022 Oct 22;23(21):12739. doi: 10.3390/ijms232112739 (PMC9655878; doi:10.3390/ijms232112739)
Supplement: Supplementary file 1 [file ijms-23-12739-s001.zip › ijms-1969469-supplementary.pdf]

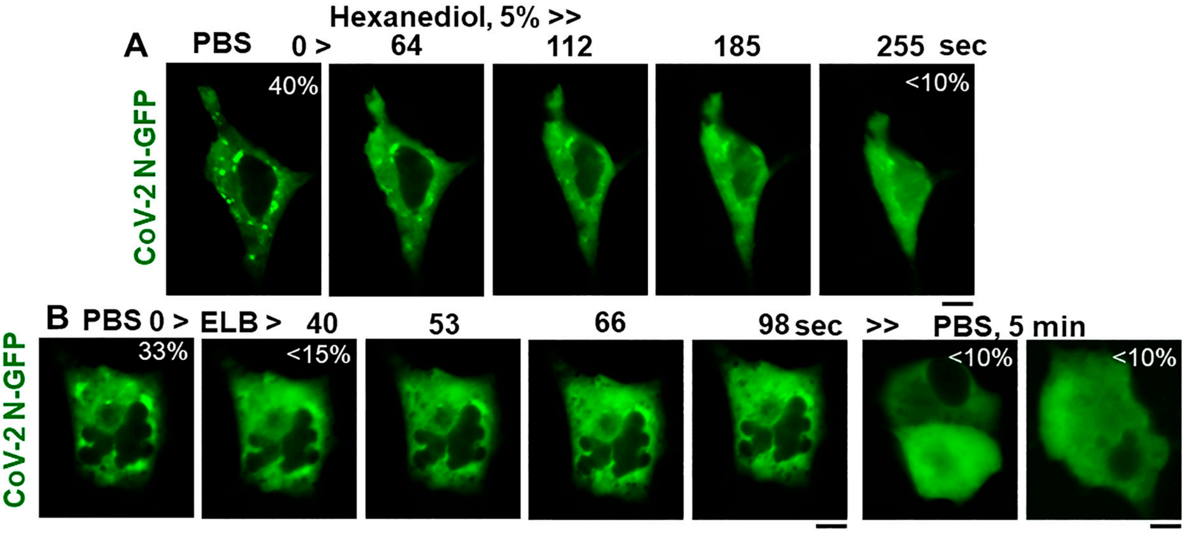

Figure S1. Properties of condensates formed by SARS-CoV-2 N-GFP. A549 cells in 35 mm plates were transiently transfected with an expression vector for SARS-CoV-2 N-GFP (Sino Biologicals) and the formation and properties of N-GFP condensates evaluated 2 days later. Panel (**A**), Live-cell time-lapse images of condensates in a cell in isotonic PBS, and after shifting to 5% hexanediol in PBS. Panel (**B**), Live-cell time-lapse images of condensates in a cell in isotonic PBS, and after shifting to hypotonic ELB; N-GFP-expressing cells in the same culture were also imaged 5 min after a further shift to isotonic PBS. All scale bars = 10 μm.

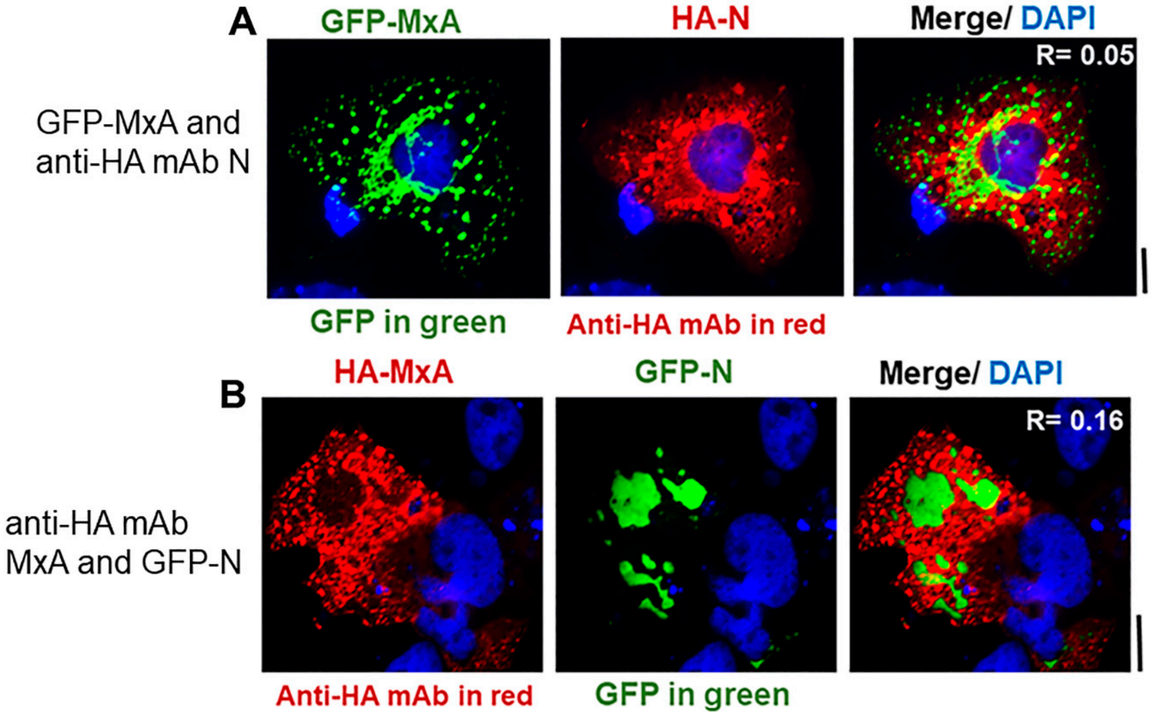

Figure S2. MxA and SARS-CoV-2 N protein form distinct condensates in the same cell. Huh7 cells in 35 mm plates were transiently transfected with pairwise combinations of respective GFP-tagged or HA-tagged vectors for MxA or SARS-CoV-2 N protein as indicated. The cultures were fixed (4% PFA) two days later, immunostained for HA (in red), and condensate formation by MxA and N protein evaluated by fluorescence imaging in green (for GFP) and

red (for HA). R is the Pearson's correlation coefficient between red and green; all scale bars = 10 μm.

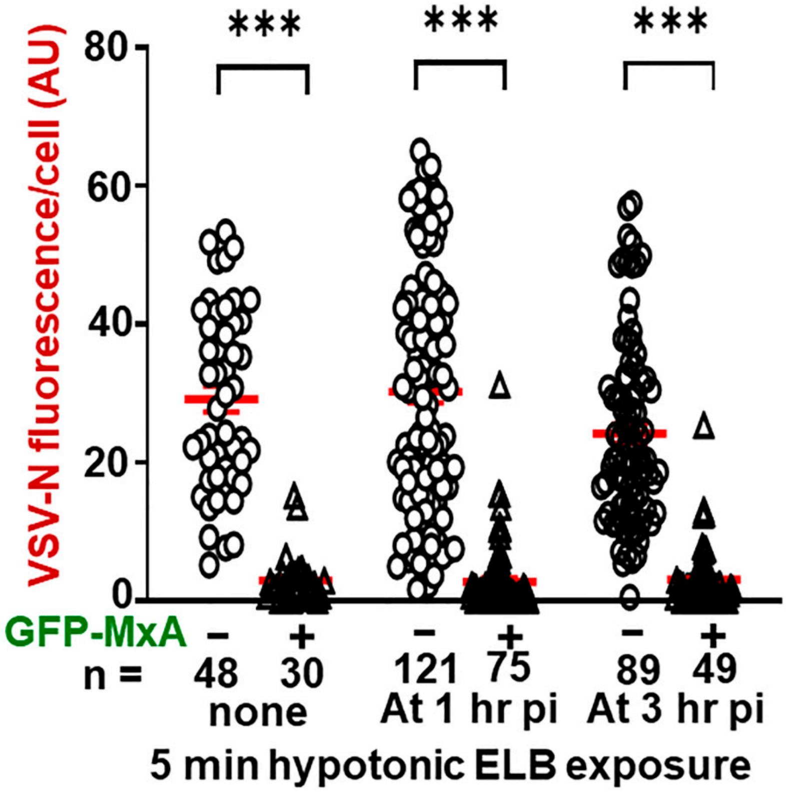

Figure S3. Antiviral activity of GFP-MxA against VSV in cultures subjected to one 5 min cycle of hypotonic disassembly/reassembly. Huh7 cells in 35 mm plates were transfected with the GFP-MxA vector, and 1 day later were infected with VSV (moi >10 pfu/cell) (13, 15). At one and 3 h after the beginning of the virus infection ("post-infection", indicated as pi in figure), respective cultures were shifted to hypotonic ELB for 5 min and then back to full medium. Live-cell microscopy confirmed the disassembly of GFP-MxA condensates when cell were shifted to ELB (not shown). All cultures were fixed at 4 h pi, and immunostained for VSV N protein (in red) (as in Refs. [44,46]). The VSV N protein fluorescence was quantitated in individual cells within the same culture without and with GFP-MxA (using Image J). n = number of cells evaluated per group in this experiment; horizontal red lines within each group indicate Mean ±SE. Statistical significance was evaluated using one-way ANOVA (Kruskal–Wallis with Dunn's post hoc test for multiple comparisons); *** $p < 0.001$.

Video S1. Spontaneous reassembly of GFP-MxA condensates in an A549-hACE2 lung-cancer cell in one-fourth strength culture medium. Movie shows time-lapse imaging (5 s/frame) of an A549-hACE2 cell expressing GFP-MxA condensates (three days after transfection) was commenced upon changing to one-fourth strength hypotonic culture medium. (Video S1 uploaded separately).
